# A Rare Cause of Pediatric Small Bowel Obstruction: Congenital Rectouterine Hernia

**DOI:** 10.7759/cureus.116097

**Published:** 2026-09-11

**Authors:** Aditya Kotla, Brandon C Toliver, Alan F Utria

**Affiliations:** 1 Pediatric Surgery, University of Iowa, Iowa City, USA; 2 Surgery, University of Iowa Hospitals & Clinics, Iowa City, USA

**Keywords:** adolescent, internal hernia, pediatrics, peritoneal defect, pouch of douglas, rectouterine pouch, small bowel obstruction

## Abstract

Small bowel obstruction caused by herniation through a peritoneal defect in the rectouterine pouch is an exceptionally rare entity, particularly in pediatric patients. We report the case of a 17-year-old previously healthy, nulliparous female who presented with four days of nausea, vomiting, and diffuse abdominal pain. She reported prior self-resolving episodes of similar symptoms, raising the possibility of intermittent herniation. CT demonstrated a high-grade small bowel obstruction with dilated loops in the pelvis and ascitic fluid, though specific signs of internal hernia, such as the mesenteric swirl sign or closed-loop configuration, were absent. Following a failed Gastrografin challenge, diagnostic laparoscopy was performed. Intraoperatively, a segment of small bowel was found incarcerated within a peritoneal defect in the rectouterine pouch. The bowel was successfully reduced laparoscopically and appeared viable. The hernia sac was excised, and the defect was widened to eliminate the potential space and prevent recurrence. The patient recovered uneventfully, with return of bowel function on postoperative day 1 and discharge on postoperative day 2. Pathologic examination confirmed fibroadipose tissue with reactive mesothelial lining consistent with a hernia sac. Given the patient’s young age, nulliparity, and absence of prior pelvic surgery, a congenital etiology is favored, though this remains presumptive. Rectouterine pouch hernias are difficult to diagnose preoperatively because their presentation is nonspecific and imaging findings are often subtle, although a pelvic transition point may provide a clue. Definitive diagnosis is typically made intraoperatively. Prompt surgical intervention is essential to prevent bowel ischemia or strangulation, and laparoscopy provides both diagnostic and therapeutic benefit. To our knowledge, this represents the second reported pediatric case of a rectouterine pouch hernia and highlights the importance of considering rare internal hernias in adolescents presenting with bowel obstruction. Early minimally invasive exploration can facilitate timely diagnosis and effective management while minimizing morbidity.

## Introduction

Internal hernias are rare causes of bowel obstruction, accounting for approximately 0.2-6% of cases [1,2]. Among the subtypes of internal hernias, herniation through a peritoneal defect in the rectouterine pouch represents an exceedingly rare entity. The existing literature on rectouterine pouch hernias consists predominantly of case reports describing multiparous, adult, and elderly women, with risk factors such as prior pelvic or abdominal surgery [3,4].

The rectouterine pouch (Pouch of Douglas) is the most dependent recess of the peritoneal cavity, situated between the uterus anteriorly and the rectum posteriorly [5]. A congenital or acquired defect in the peritoneum lining this pouch can result in internal herniation of bowel. The anatomy of the defect and its location deep in the pelvis mean that it often lacks the classic radiographic hallmarks of an internal hernia, and subsequently, the diagnosis is rarely suspected preoperatively.

To date, only one pediatric case of a rectouterine pouch hernia has been published: a 17-year-old female patient in 1988 [6]. Here, we present the case of a pediatric patient who presented with a small bowel obstruction, was managed operatively, and was found to have a rectouterine pouch hernia.

## Case presentation

A 17-year-old otherwise healthy, nulliparous female presented to the pediatric ED as a transfer from an outside hospital with four days of nausea, vomiting, and generalized abdominal pain. She was in her normal state of health prior to the onset of symptoms and did not recall a specific trauma or inciting event that preceded the development of these symptoms. She had no significant past medical history. Her past surgical history was significant for a laparoscopic appendectomy for uncomplicated appendicitis performed at age 13. She reported similar prior episodes of abdominal pain with nausea and emesis that had self-resolved within 24 hours. Due to her persistent symptoms, she presented to an outside hospital ED, where a nasogastric tube was placed for decompression, laboratory tests were obtained, and a CT abdomen and pelvis with IV contrast was performed. She was subsequently transferred to our center for pediatric surgery evaluation and management.

On evaluation, her vital signs were appropriate for age, and she was in no acute distress. Physical examination was significant for tenderness to deep palpation in all four quadrants, mild distention, and no guarding or peritonitis. Laboratory tests were unremarkable, including a white blood cell count of 9.3 × 10³/µL and a comprehensive metabolic panel within normal limits.

Urine pregnancy testing was negative. Review of the CT obtained at the outside hospital demonstrated a small amount of free fluid in the pelvis, a surgically absent appendix, and a high-grade small bowel obstruction with concern for a transition point in the right lower quadrant (Figure 1, 2). There was no evidence of bowel distress or twisting of the mesenteric vessels (swirl sign).

**Figure 1:**
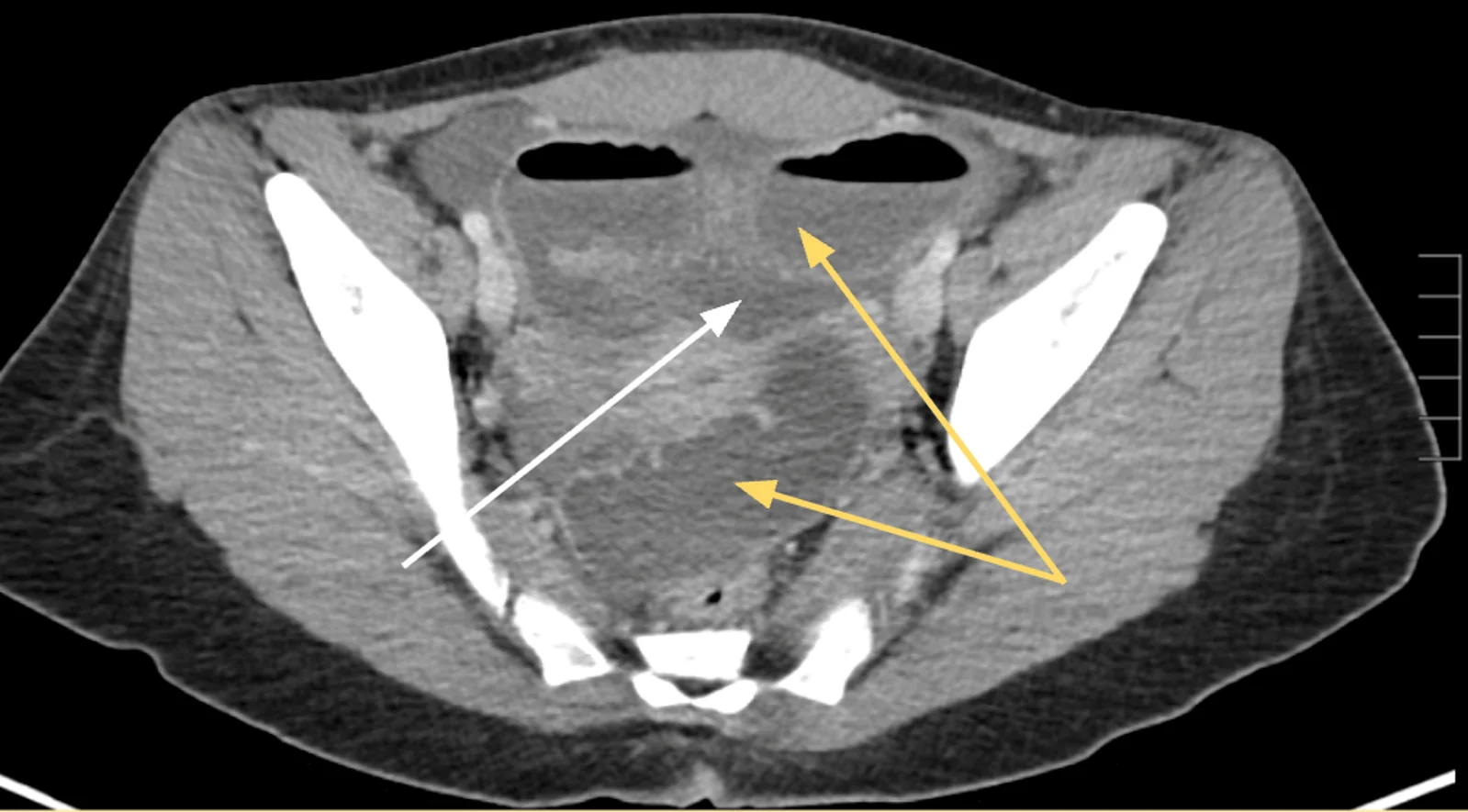
CT abdomen and pelvis axial slice. This CT abdomen and pelvis axial slice demonstrates significantly dilated bowel in the inferior pelvis (yellow arrows) and ascitic fluid (white arrow). Specific signs of internal hernia, including the mesenteric swirl sign and closed-loop configuration, are not identified on this image.

**Figure 2:**
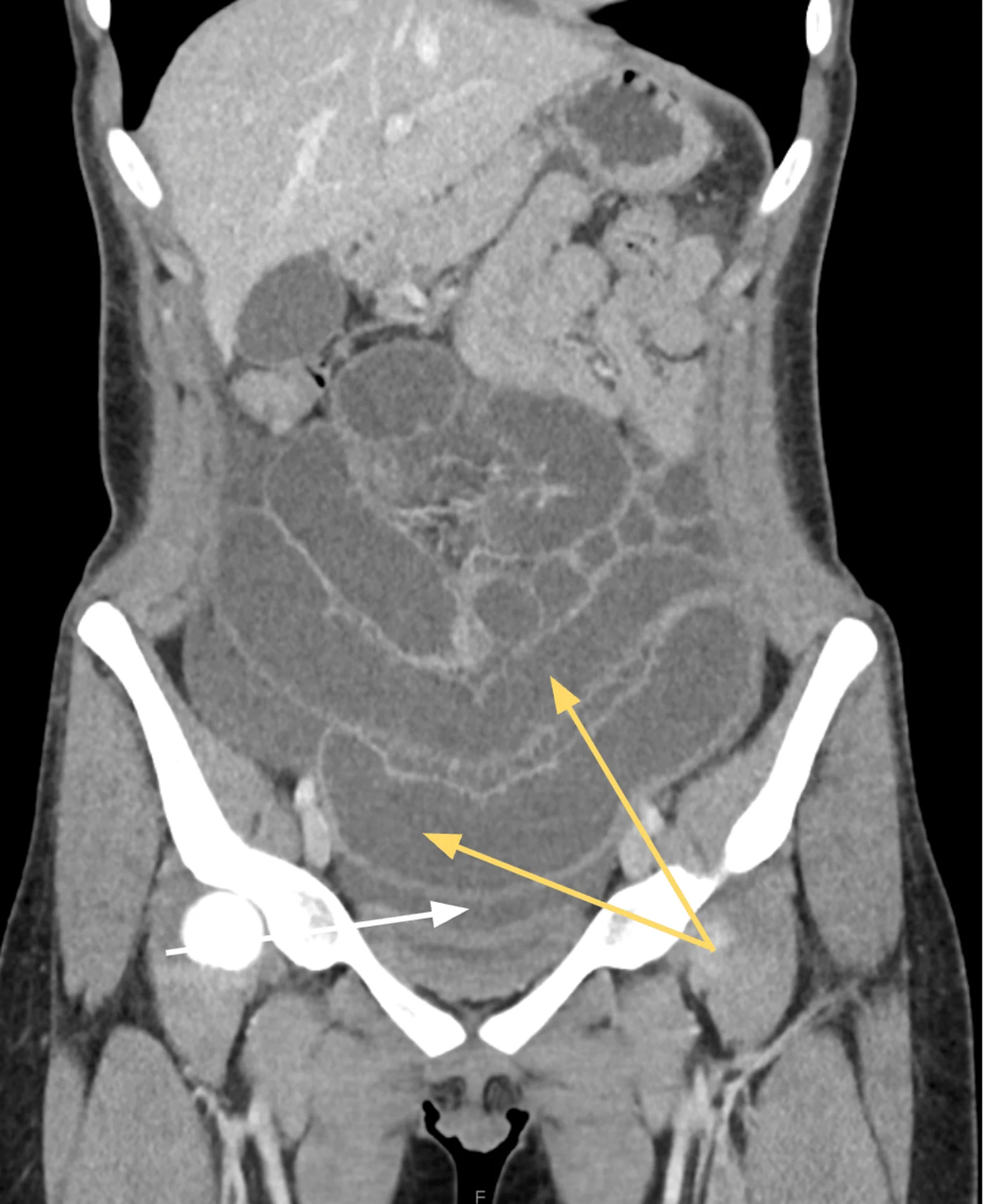
CT abdomen and pelvis coronal slice. This CT abdomen and pelvis coronal slice demonstrates dilated small bowel loops (yellow arrows) and ascitic fluid between bowel (white arrow) extending to the lower pelvis.

The leading diagnosis was adhesive small bowel obstruction related to her prior appendectomy. Other considerations included internal hernia, pelvic inflammatory disease, and endometriosis. Given her stable clinical status and the absence of concerning radiographic features (swirl sign, bowel compromise, or closed-loop obstruction), she was admitted and underwent a water-soluble contrast challenge via the nasogastric tube [7]. Serial abdominal radiographs at four and eight hours demonstrated no passage of contrast into the colon (Figure 3). Operative intervention was therefore pursued.

**Figure 3:**
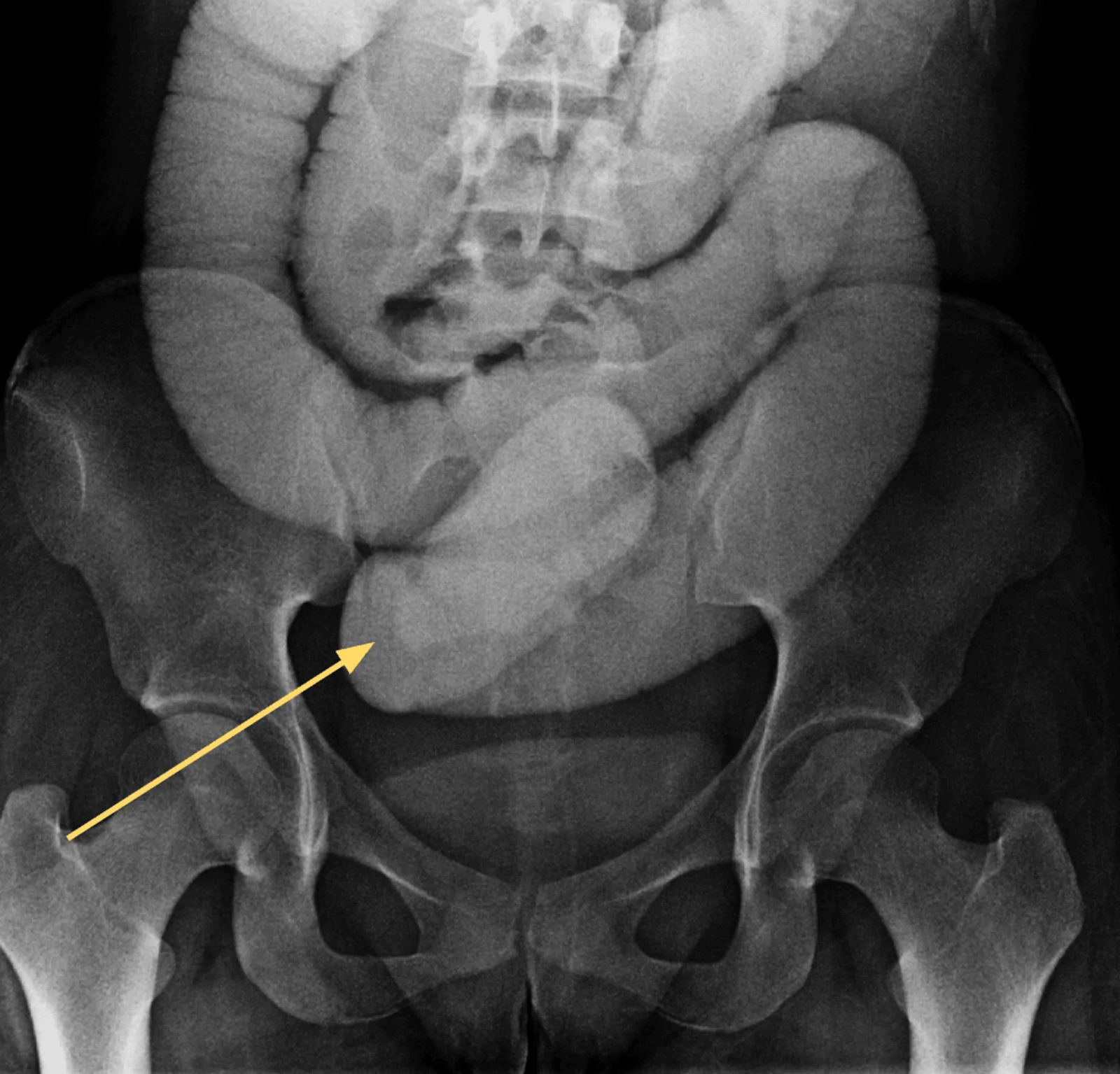
Water-soluble Gastrografin challenge eight hours post-administration. Eight-hour water-soluble contrast challenge radiograph demonstrating contrast pooling in dilated small bowel loops within the pelvis (arrow), with failure of contrast to reach the colon, consistent with a transition point in the pelvis.

Operative technique 

Diagnostic laparoscopy revealed significant dilation of the small bowel with decompressed small bowel at the level of the terminal ileum. The appendix was surgically absent, and there were no adhesions in the right lower quadrant. The small intestine was run in a retrograde fashion from the decompressed terminal ileum. A knuckle of distal ileum was noted to be incarcerated within a peritoneal defect in the rectouterine pouch (Figure 4). The bowel proximal to this incarcerated segment appeared distended. Using gentle traction, we were able to reduce the incarcerated bowel from the hernia. The reduced bowel appeared viable. Inspection of the hernia sac did not reveal any additional concerning contents. We opted to excise the hernia sac for pathologic evaluation and opened the defect widely to prevent further incarceration. The remainder of the abdomen was evaluated, with no other concerning findings.

**Figure 4:**
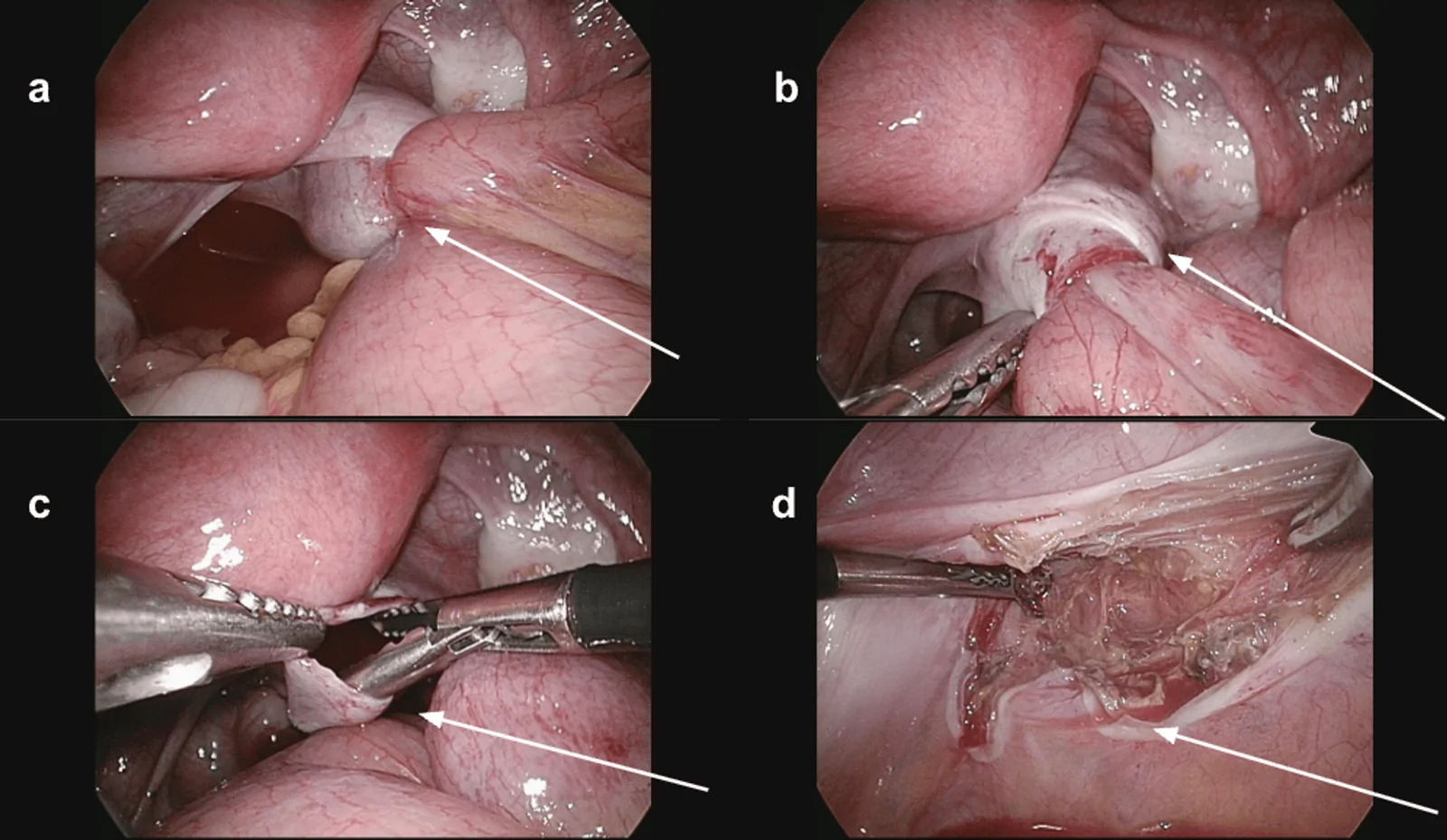
Laparoscopic operative images. This figure consists of laparoscopic operative images showing encountering the transition point after running the small bowel (a), initiation of reduction of the incarcerated bowel through gentle traction (b), the defect in the rectouterine pouch after release of the incarcerated bowel (c), and widely opened defect (d).

Postoperative course 

On postoperative day 1, she had return of bowel function and decreased nasogastric tube output. The nasogastric tube was removed, and a clear liquid diet was initiated. She continued to progress well, and on postoperative day 2, she was advanced to a general diet and discharged home. Pathologic examination of the tissue biopsy demonstrated fibroadipose tissue with reactive mesothelial lining and chronic inflammation, consistent with a hernia sac. There was no evidence of endometriosis. At six-week follow-up, she remained asymptomatic without complications or recurrence and was discharged from clinic.

## Discussion

The rectouterine pouch is also known as the Pouch of Douglas, named after the Scottish anatomist James Douglas (1675-1742). It is the most dependent recess of the peritoneal cavity, located between the uterus anteriorly and the rectum posteriorly [5]. This anatomical space exhibits extensive variation in depth among individuals, ranging from 11% to 89% of total vaginal length in healthy women, with deeper pouches potentially predisposing to pelvic floor disorders and prolapse [8]. While this location is commonly involved in gynecologic conditions such as abscesses, cysts, and endometriosis, internal herniation through a defect in this area is exceedingly rare [5].

The etiology of rectouterine pouch hernias can be classified as either congenital or acquired. Acquired defects are associated with multiparity or prior pelvic surgery, particularly hysterectomies, which explains the predominance of cases reported in elderly women [3,4,9]. Pelvic inflammatory disease and endometriosis are known risk factors for internal hernias through the broad ligament. Although not reported in the literature, these conditions could theoretically also predispose patients to hernias in the rectouterine space [10,11]. The embryologic basis of congenital peritoneal defects remains incompletely understood [1].

In the present case, the absence of predisposing risk factors for an acquired hernia supports a congenital origin. Notably, only one other pediatric case has been documented in the literature, published in 1988 [6]. Our patient had experienced previous self-limited episodes of abdominal pain, nausea, and vomiting, consistent with intermittent obstruction at the site of the hernia [12,13]. These episodes had all self-resolved within 24 hours after the patient decreased her oral intake due to nausea and emesis.

Preoperative diagnosis remains challenging. Clinical findings typically reflect small bowel obstruction without distinguishing features. CT is the preferred imaging modality for a suspected internal hernia and may demonstrate clustered small bowel loops, closed-loop obstruction, or mesenteric vessel convergence (swirl sign) [1,13-15]. However, as demonstrated by our case, while CT can clearly demonstrate a high-grade bowel obstruction, it can often fail to demonstrate the etiology. Therefore, diagnosis may only be established at the time of surgical exploration [3].

The definitive management for a high-grade bowel obstruction is surgical. Prompt surgical intervention is essential to prevent complications including bowel ischemia, strangulation, necrosis, and perforation. Minimally invasive laparoscopic techniques allow for both diagnostic confirmation and definitive repair [3,9]. Surgical treatment involves careful reduction of the herniated bowel loops, followed by either primary closure of the peritoneal defect or deliberate widening/enlargement of the defect to eliminate the potential space and reduce the risk of recurrence. The choice between these approaches depends on the anatomic context. The anatomy of a rectouterine pouch defect differs fundamentally from a mesenteric defect, where closure is indicated [16]. With peritoneal defects, widening the defect and excising the peritoneum eliminates the potential for re-herniation and is technically straightforward. The excised peritoneum should be sent to pathology and examined for evidence of endometriosis [10].

Given that only one prior pediatric case has been described, our report meaningfully expands the limited literature and reinforces the importance of maintaining a broad differential diagnosis in pediatric small bowel obstruction. Heightened awareness of this rare entity may facilitate timely operative management and prevent avoidable complications.

## Conclusions

Rectouterine pouch hernias are exceptionally rare causes of internal hernias and small bowel obstructions, with only one previously reported pediatric case in the literature. The clinical presentation is nonspecific, and preoperative imaging may demonstrate pelvic transition points without definitively identifying the underlying defect. As a result, the diagnosis is established at the time of operative intervention.

Our case expands on the limited pediatric literature and prompts consideration of internal hernias of the rectouterine pouch in female pediatric patients presenting with small bowel obstruction and a transition point in the pelvis. Early surgical intervention facilitates both definitive diagnosis and repair, thereby reducing the risk of morbidity. Increased awareness of this rare entity may improve timely recognition and outcomes.
